# Establishing a sensitive fluorescence-based quantification method for cyclic nucleotides

**DOI:** 10.1186/s12896-020-00633-y

**Published:** 2020-08-27

**Authors:** Nadine Gruteser, Viktoria Kohlhas, Sabine Balfanz, Arne Franzen, Anne Günther, Andreas Offenhäusser, Frank Müller, Viacheslav Nikolaev, Martin J. Lohse, Arnd Baumann

**Affiliations:** 1 Institute of Biological Information Processing (Molecular and Cellular Physiology, IBI-1), Forschungszentrum Jülich, 52428 Jülich, Germany; 2 Present address: CECAD Research Center, 50931 Cologne, Germany; 3 Present address: RIKEN Center for Brain Science, Wako, Saitama, 351-0198 Japan; 4 Institute of Biological Information Processing (Bioelectronics, IBI-3), Forschungszentrum Jülich, 52428 Jülich, Germany; 5 Institute of Experimental Cardiovascular Research, University Medical Center Hamburg-Eppendorf, 20246 Hamburg, Germany; 6 Institute of Pharmacology and Toxicology, University of Würzburg, 97078 Würzburg, Germany; 7 Max Delbrück Center for Molecular Medicine, 13125 Berlin, Germany

**Keywords:** Cyclic nucleotide quantification, Cell-based assay, Epac1-camps, Optogenetic sensor, Signaling

## Abstract

**Background:**

Approximately 40% of prescribed drugs exert their activity via GTP-binding protein-coupled receptors (GPCRs). Once activated, these receptors cause transient changes in the concentration of second messengers, e.g., cyclic adenosine 3′,5′-monophosphate (cAMP). Specific and efficacious genetically encoded biosensors have been developed to monitor cAMP fluctuations with high spatial and temporal resolution in living cells or tissue. A well characterized biosensor for cAMP is the Förster resonance energy transfer (FRET)-based Epac1-camps protein. Pharmacological characterization of newly developed ligands acting at GPCRs often includes numerical quantification of the second messenger amount that was produced.

**Results:**

To quantify cellular cAMP concentrations, we bacterially over-expressed and purified Epac1-camps and applied the purified protein in a cell-free detection assay for cAMP in a multi-well format. We found that the biosensor can detect as little as 0.15 pmol of cAMP, and that the sensitivity is not impaired by non-physiological salt concentrations or pH values. Notably, the assay tolerated desiccation and storage of the protein without affecting Epac1-camps cyclic nucleotide sensitivity.

**Conclusions:**

We found that determination cAMP in lysates obtained from cell assays or tissue samples by purified Epac1-camps is a robust, fast, and sensitive assay suitable for routine and high throughput analyses.

## Background

Signaling within and between cells often invokes transient and spatially restricted changes of intracellular second messenger concentrations. In addition to calcium (Ca^2+^) signals, changes in intracellular concentrations of cyclic adenosine 3′,5′-monophosphate (cAMP) or cyclic guanosine 3′,5′-monophosphate (cGMP) play an important role in cell signaling. As second messengers, cyclic nucleotides are known to control the activity of protein kinases and phosphatases as well as Popeye domain containing (Popdc [6];) proteins. Kinases and phosphatases then modulate the phosphorylation status of downstream proteins participating, e.g., in cellular trafficking, metabolic uptake mechanisms as well as regulation of gene transcription (reviewed in: [22, 37]). Furthermore, cyclic nucleotides have been shown to either directly activate cyclic nucleotide-gated ion channels in many cells, including photoreceptors and sensory neurons (reviewed in: [3, 21]) or to shift the voltage-dependent activation of hyperpolarization-activated and cyclic nucleotide-gated ion channels in neurons and cardiac tissue (reviewed in: [4, 18, 20]).

To gain insight into cellular second messenger dependent signaling, several strategies can be followed to quantify second messenger concentrations under different experimental conditions. Mean values of such concentrations can be obtained when examining cell or tissue lysates. Typically, competition binding experiments are performed where the compounds present in the lysate compete with a radiolabeled substrate for a binding site present on a specific binding protein [9, 16] or an antibody [13]. Alternatively, immunoassays have been developed where compounds, e.g. cAMP, present in the lysate compete with chemically modified cAMP molecules [17] for a binding site on a specific antibody. Detection occurs via another set of antibodies that recognize the modified but not the native compound. These secondary antibodies typically are coupled to an enzyme that metabolizes a substrate which then emits luminescence [11]. A large community is applying these strategies to quantify cyclic nucleotide concentrations upon exposing cells or tissue to different external conditions as well as in high throughput screening assays. However, a drawback of many of these approaches is a relatively long hands-on time which, in some cases, requires overnight incubation steps.

In recent years, the development of genetically encoded sensors has revolutionized the field and allowed to measure changes in second messenger concentrations with high spatial and temporal resolution at the cellular level [15, 24, 27, 31]. One well-known detector of cAMP is Epac1-camps [26]. This protein allows Förster resonance energy transfer (FRET)-based measurements in single cells. It harbors a central cAMP-binding domain originating from Epac1 [12] that is N-terminally fused to an enhanced yellow fluorescent protein (EYFP) and C-terminally linked to an enhanced cyan fluorescent protein (ECFP). In the absence of cAMP both fluorescent proteins are in close contact. Thus, when the donor fluorophore ECFP is excited at 430 nm, the acceptor fluorophore EYFP is excited via FRET and emits fluorescence that can be measured at 530 nm. Once the intracellular cAMP concentration increases, cAMP binds to the Epac1 domain of the sensor. This induces a conformational change by which the ECFP and EYFP moieties move away from each other. Consequently, upon ECFP excitation less energy is transferred to EYFP, and the EYFP emission decreases whereas simultaneously the ECFP emission (475 nm) increases [26].

Since we are interested in determining changes in cyclic nucleotide concentrations not only in individual cells, we decided to examine the applicability of Epac1-camps to substitute for currently available detection methods to quantify second messengers like cAMP. To achieve this goal, we expressed Epac1-camps in *E.coli* cells and purified the protein by Ni-NTA- and size-exclusion chromatography. Purified protein was assessed for sensitivity and specificity in 96 multi-well plates (MWPs) by adding concentration series of cAMP or cGMP. Changes of the sensor’s fluorescence were registered in a plate reader. With EC_50_ values of 1.3 μM (cAMP) and 7.8 μM (cGMP), Epac1-camps showed preferred interaction with cAMP. Yet, interaction with cGMP is not negligible. Under the chosen experimental conditions, the threshold amount for cAMP detection was ≅ 0.15 pmol. The functionality of the assay was not affected by different salt concentrations or pH values. Notably, purified Epac1-camps protein could be dried in the MWP and stored at 4 °C until further use. After re-constitution, the sensors’ activity was fully preserved. Based on the sensitivity, stability, as well as short handling and incubation times, the assay is equivalent if not advantageous in comparison to currently available methods.

## Results

### Expression and purification of Epac1-camps-His_6_

A C-terminally His_6_-tagged Epac1-camps cDNA was cloned in pET11a expression vector. Protein expression was performed with BL21(DE3) plysS and BL21(DE3)CodonPlus-RIL *E.coli* strains. For induction of protein expression, a final concentration of 1 mM IPTG was applied and culture growth was continued at 37 °C for 4 h, at 30 °C overnight, and at 20 °C overnight. The highest expression rate was with BL21(DE3)-CodonPlus-RIL cells and at 30 °C with overnight expression. After cell lysis the Epac1-camps-His_6_ protein was enriched by Ni-NTA affinity chromatography. Typically, up to 10 mg protein were purified from a 500 ml expression culture. Fractions containing the sensor protein were combined and subjected to size exclusion chromatography to further purify the sample. Aliquots from eluate fractions were analysed by SDS-PAGE. The combined strategy of Ni-NTA affinity and size exclusion chromatography resulted in highly pure and homogenous preparations of Epac1-camps-His_6_, which was advantageous to establishing the cyclic nucleotide quantification assay.

### Interaction of Epac1-camps-His_6_ and cyclic nucleotides

**Fig. 1:**
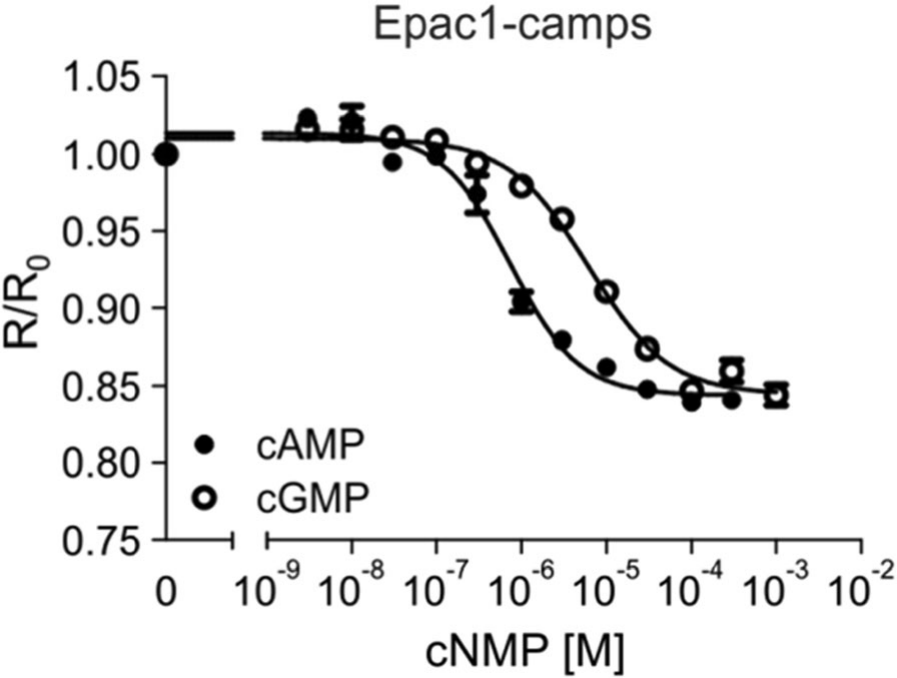
Characterization of Epac1-camps-His_6_ in multi-well plates. Purified Epac1-camps-His_6_ protein was diluted to a concentration of 0.7 μM in IS buffer and 90 μl of the sample was added to each well of a 96 multi well plate. Excitation was at 430 nm and emission was recorded at 475 nm (ECFP) and 530 nm (EYFP). After recording the basal fluorescence for ECFP and EYFP, increasing concentrations of cAMP or cGMP were added to each well. The final volume was 100 μl and final concentrations of cyclic nucleotides ranged from 10^− 9^ - 10^− 4^ M. The EYFP/ECFP ratio (R) for each well was calculated and normalized to the ratio of the basal fluorescence in the absence of cyclic nucleotides (R_0_). Normalized data were plotted against cyclic nucleotide concentrations. Each data point is given as mean value (±SD) from four identically treated wells of a representative experiment. EC_50_ values were calculated with a four-parameter nonlinear regression using GraphPad Prism v5.04

Binding of cyclic nucleotides results in a change of the fluorescence emission of the Epac1-camps FRET sensor [26]. To assess the functionality of the purified Epac1-camps-His_6_ protein, the protein was diluted to 0.7 μM and 90 μl of this solution was added to each well of a 96 multi well plate (MWP). Fluorescence emission in each well was measured in a plate reader. Excitation was at 430 nm and emission was recorded at 475 nm (ECFP) and 530 nm (EYFP). After recording the basal fluorescence for ECFP and EYFP, series of increasing concentrations of cAMP or cGMP were added to each well. The final volume in each well was 100 μl, and final concentrations of cyclic nucleotides ranged from 10^− 9^ - 10^− 4^ M. Incubation of the sensor protein with its ligands was for 30 min. at room temperature. Then, the EYFP/ECFP emission ratio (R) for each well was determined and normalized to the emission ratio of the basal fluorescence, i.e., in the absence of cAMP or cGMP (R_0_). Normalized data were plotted against cyclic nucleotide concentrations and EC_50_ values were calculated. In Fig. 1 a representative measurement is shown. Mean values with standard deviations were obtained from four-fold determinations for each cyclic nucleotide. With increasing concentrations of cAMP and cGMP the FRET-based emission of EYFP decreases. The responses saturated at ≥10^− 5^ M (cAMP) and ≥ 3 × 10^− 5^ M (cGMP). The EC_50_ values obtained from these concentration response curves for cAMP and cGMP were 1.3 × 10^− 6^ M and 7.8 × 10^− 6^ M, respectively. Similar results were obtained in at least three independent measurements performed on two protein preparations that were independently expressed and purified.

### Measurements with different salt concentrations

**Fig. 2:**
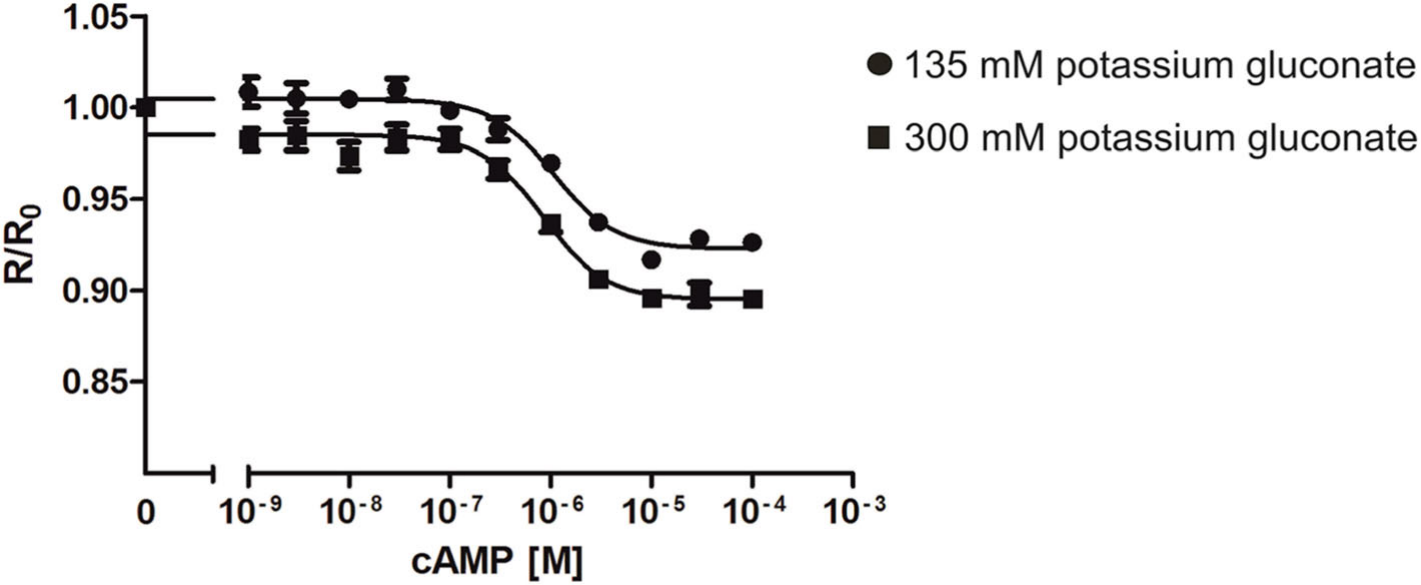
Change of cAMP-dependent Epac1-camps-His_6_ fluorescence at different salt concentrations. For measurements depicted in this graph, an independently expressed and purified Epac1-camps sample has been used (c.f. Figure 1). Purified Epac1-camps-His_6_ protein was diluted to a concentration of 0.7 μM in IS buffer containing 135 mM (•) or 300 mM (▅) potassium gluconate. 90 μl of each sample was added to 48 wells of a 96 multi well plate allowing simultaneous four-fold measurements. Excitation was at 430 nm and emission was recorded at 475 nm (ECFP) and 530 nm (EYFP). After recording the basal fluorescence for ECFP and EYFP, increasing concentrations of cAMP were added. The EYFP/ECFP ratio (R) for each well was calculated and normalized to the ratio of the basal fluorescence in the absence of cAMP (R_0_). Normalized data (mean values ± SD) were plotted against cAMP concentrations. EC_50_ values were calculated with a four-parameter nonlinear regression using GraphPad Prism v5.04

In the experiments depicted in Fig. 1, cyclic nucleotides were added in regular IS buffer. Under experimental conditions, however, cyclic nucleotides originating from cell or tissue extracts may contain higher or different salt concentrations. Therefore, we performed a similar assay as shown in Fig. 1 and used IS buffer containing 135 mM or 300 mM potassium gluconate. The results are shown in Fig. 2. The behavior of the sensor protein to cAMP was very similar for both salt concentrations. The EC_50_ determined at 300 mM potassium gluconate is slightly shifted to a lower value of 8.4 × 10^− 7^ M compared to 1.0 × 10^− 6^ M determined in regular IS buffer. Thus, the sensitivity of purified Epac1-camps-His_6_ protein was rather stable which would allow measuring samples containing different salt compositions. However, rather than to directly compare results at physiological (135 mM) or high (300 mM) salt concentrations the experiments were performed to test Epac1-camps capability to register cAMP amounts at different ionic strengths. Thus, it is vital to establish the calibration curve and to conduct sample measurements using identical buffer conditions.

### Measurements at different pH values

**Fig. 3:**
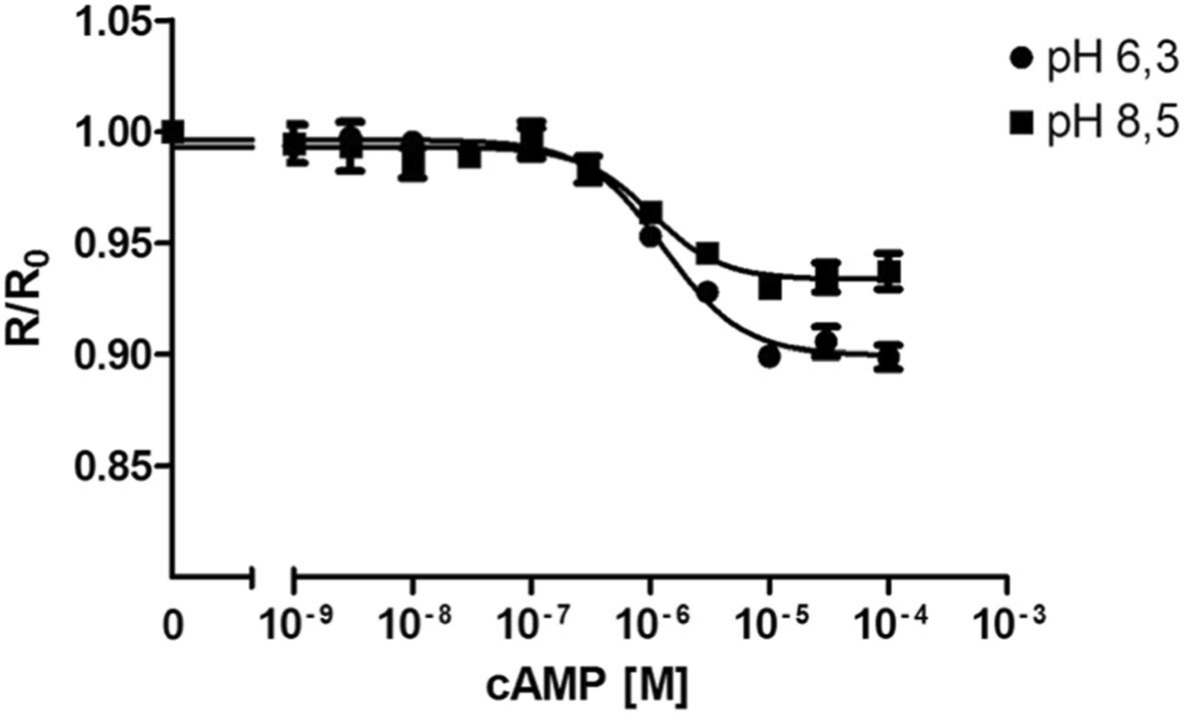
Change of cAMP-dependent Epac1-camps-His_6_ fluorescence at different pH values. Purified Epac1-camps-His_6_ protein was diluted to a concentration of 0.7 μM in IS buffer with pH 6.3 or 8.5. 90 μl of each sample was added to 48 wells of a 96 multi well plate allowing simultaneous four-fold measurements. Excitation was at 430 nm and emission was recorded at 475 nm (ECFP) and 530 nm (EYFP). After recording the basal fluorescence for ECFP and EYFP, increasing concentrations of cAMP were added. The EYFP/ECFP ratio (R) for each well was calculated and normalized to the ratio of the basal fluorescence in the absence of cAMP (R_0_). Normalized data (mean values ± SD) were plotted against cAMP concentrations. EC_50_ values were calculated with a four-parameter nonlinear regression using GraphPad Prism v5.04. A representative of two independently performed experiments is shown

Similar to the experiments performed with different salt concentrations, the impact of buffers with different pH on Epac1-camps-His_6_ to register cAMP concentrations was examined. Here, experiments were conducted in IS buffer at pH 6.3 and 8.5. All previous measurements were in IS buffer at pH 7.4. The result is shown in Fig. 3. Protein was diluted in the respective buffer (pH 6.3 or 8.5) and transferred into 48 wells of a 96 MWP, allowing four-fold determinations for the cAMP concentration series. Under both conditions the cAMP-dependent response of Epac1-camps-His_6_ was very similar reaching saturation at cAMP concentrations ≥10^− 5^ M. Notably, EC_50_ values of 1.3 × 10^− 6^ M (pH 6.3) and 1.0 × 10^− 6^ M (pH 8.5) were almost identical to those determined in IS buffer at pH 7.4 (see Fig. 1). Therefore, samples deviating by at least one order of magnitude to the acidic or alkaline range from the physiological pH (≈7.4) can be applied and examined. However, since GFP and its mutants are known to be sensitive to pH (see, e.g., [8, 23]), it is necessary to calibrate and to perform each measurement at the same pH value.

### Measurements with desiccated and re-constituted sensor protein

**Fig. 4:**
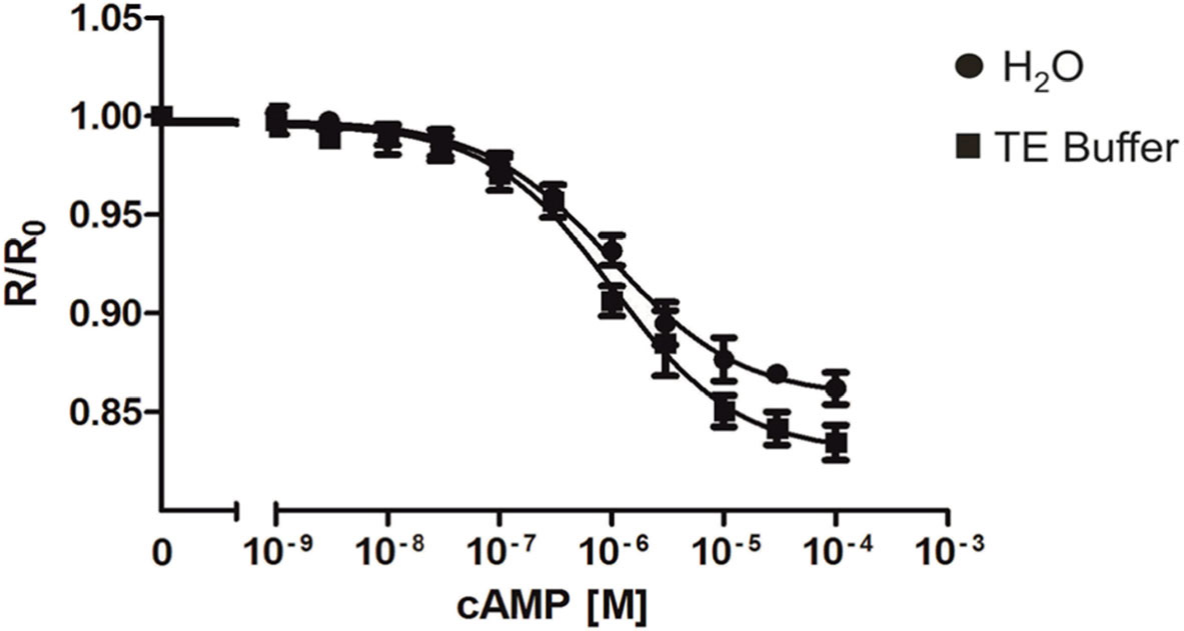
Change of cAMP-dependent Epac1-camps-His_6_ fluorescence using desiccated and re-constituted samples. Purified Epac1-camps-His_6_ protein was diluted to a concentration of 0.7 μM in IS buffer, transferred to all wells of a 96 MWP, and desiccated in a fridge. Protein in 48 wells was reconstituted in bi-destilled H_2_O and protein in the remaining wells was reconstituted in TE-buffer. This design allowed simultaneous four-fold measurements. Excitation was at 430 nm and emission was recorded at 475 nm (ECFP) and 530 nm (EYFP). After recording the basal fluorescence for ECFP and EYFP, increasing concentrations of cAMP were added. The EYFP/ECFP ratio (R) for each well was calculated and normalized to the ratio of the basal fluorescence in the absence of cAMP (R_0_). Normalized data (mean values ± SD) were plotted against cAMP concentrations. EC_50_ values were calculated with a four-parameter nonlinear regression using GraphPad Prism v5.04. A representative of two independently performed experiments is shown

In order to gain information about stability and conceivable storage conditions of Epac1-camps-His_6_, experiments were performed on purified protein that was desiccated in 96 MWPs. As for the previous experiments the protein was diluted to 0.7 μM and 90 μl of the solution was transferred into each well of a 96 MWP. Desiccation of the protein was achieved in a fridge at 4 °C. Whether the functionality of Epac1-camps-His_6_ was affected by this treatment was assessed once the desiccated protein was re-constituted. For solubilisation of the protein, bi-distilled H_2_O was applied to 48 wells and TE buffer (10 mM Tris/HCl, pH 7.4; 1 mM EDTA) was applied to the remaining 48 wells of the plate. A concentration series of cAMP was applied and the cAMP-dependent response of Epac1-camps-His_6_ was measured. As depicted in Fig. 4 the protein was successfully re-constituted and displayed the typical cAMP-dependent decrease of the FRET signal. It is worth noting that testing samples stored desiccated for 3 weeks at 4 °C were as active as samples prepared from frozen protein stocks. As already mentioned for measurements performed at different salt concentrations or pH values, it is necessary also with desiccated samples to establish and keep experimental conditions constant. This would allow to directly comparing data sets obtained from independent series of experiments.

### Measurements with probes obtained from cell cultures

**Fig. 5:**
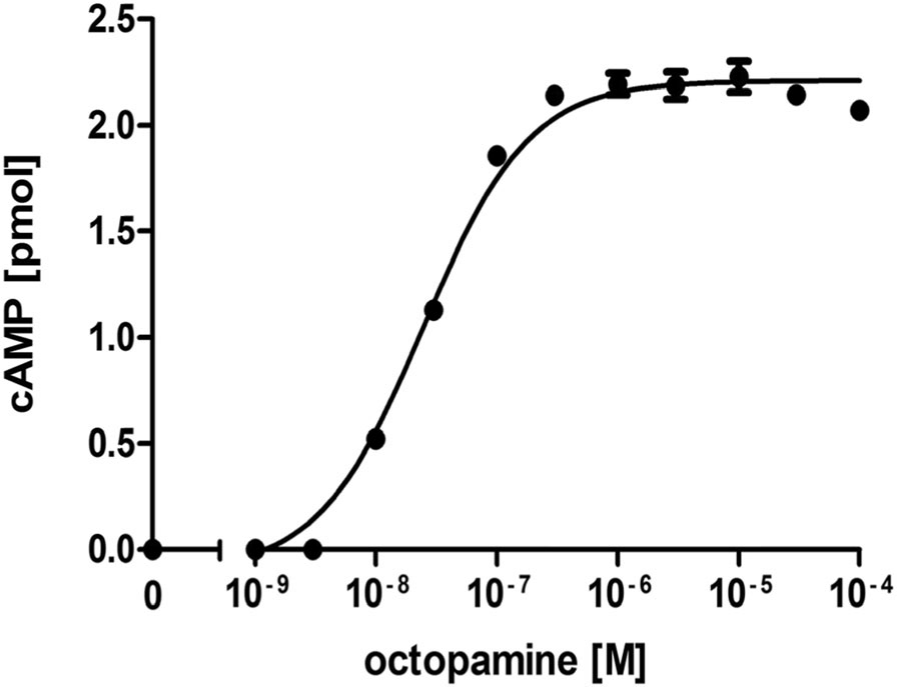
Quantification of cAMP produced by GPCR activation. A cell line constitutively expressing an octopamine receptor from *Drosophila melanogaster* (HEK293 – DmOctβ1R) was incubated with increasing concentrations (10^− 9^ – 10^− 4^ M) octopamine in a 24 MWP for 30 min at 37 °C. Cells were lyzed by adding ice-cold ethanol. Extracts were lyophilized, reconstituted in H_2_O and the amount of cAMP was determined with purified Epac1-camps-His_6_ protein as described before. Excitation was at 430 nm and fluorescence emission was recorded at 475 nm (ECFP) and 530 nm (EYFP). After recording basal fluorescence emission for ECFP and EYFP, samples were added and incubated for 30 min at room temperature. Finally, the EYFP/ECFP ratio (R) of fluorescence emission for each well was calculated and normalized to the ratio of the basal fluorescence emission (R_0_). Using a calibration curve established with known cAMP concentrations in parallel, the cAMP amount present in each sample was determined. Mean values (± SD) from quadruplicate determinations were plotted against octopamine concentrations. The EC_50_ value (2.475 × 10^− 8^ M) of DmOctβ1R was calculated with a four-parameter nonlinear regression analysis using GraphPad Prism v5.04. A representative of three independently performed experiments is shown

So far all measurements were performed with chemically pure cAMP or cGMP solutions. In order to examine the assay’s usability for cell derived probes we prepared extracts from a cell line in which cAMP production was induced by a GPCR-mediated signaling cascade. We used a previously established cell line that constitutively expresses an octopamine receptor from *Drosophila melanogaster* (DmOctβ1R [2];). Octopamine is a biogenic amine mainly present in protostomes and is a homolog of catecholamines in mammals [32]. The phenolamine binds to GPCRs that activate different intracellular signaling pathways. When α-type octopamine receptors are activated, this results in Ca^2+^ release from the endoplasmic reticulum. When β-type octopamine receptors, like DmOctβ1R, are activated, this results in cAMP production via adenylyl cyclases ([2]; see also [5]). The cell line expressing DmOctβ1R was seeded in 24 MWPs and a concentration series of octopamine (10^− 9^ – 10^− 4^ M together with 100 μM IBMX) was applied. After incubation for 30 min at 37 °C, the ligand-containing solution was aspirated and cells were lysed in ice-cold ethanol. The ethanolic suspension was lyophilized, reconstituted in H_2_O and then added to Epac1-camps-His_6_ protein in a 96 MWP as described for the previous assays. A calibration curve was established with known cAMP concentrations and the amount of cAMP in cell extracts were plotted against the octopamine concentrations. The result of a representative measurement and its derived concentration-response curve is depicted in Fig. 5. Data points (mean values ± SD) were derived from quadruplicate determinations for each octopamine concentration. The delineated EC_50_ of 2.5 × 10^− 8^ M for octopamine is very similar to previous pharmacological data determined on this receptor (≈3 × 10^− 8^ M, [2, 25]). This result demonstrates that the assay employing purified Epac1-camps-His_6_ is fast, sensitive and well suited to quantify second messenger concentrations that originate, e.g., from cell-based assays or tissue extracts to establish pharmacological and functional profiles of membrane receptors.

## Discussion

Here we used the optogenetic sensor protein Epac1-camps and established a sensitive and fast detection assay to determine cAMP concentrations originating from cellular signaling cascades. Fluorescence emission signals were read out in a plate reader using conventional 96 MWPs. With a detection limit of ≅0.15 pmol (see Fig. 1) the assay is well suited to quantify cAMP concentrations extracted from ca. 250,000 cells.

Transient and dynamic fluctuations of intracellular second messenger concentrations like cyclic nucleotides or Ca^2+^ are at the heart of cellular signaling circuits, especially in neural tissue [19]. Thus, when studying functional and physiological effects of cellular signal transduction processes, an ultimate aim is to quantify the concentration changes of these messengers upon a certain stimulus. Often, intracellular second messengers are controlled by the activity of G-protein coupled receptors [29]. In humans, this group of membrane receptors is the target of up to 40% of prescription medicines [38]. Consequently, there is ongoing interest to examine chemical libraries for candidates acting as agonists or antagonists on specific receptors to uncover beneficial new drugs and/or new drug targets. In recent years platforms have been developed allowing high throughput screening of such libraries, typically employing cell-based strategies with colorimetric read out [1, 28]. More recently, microfluidics in combination with high resolution imaging [10] has been introduced which allows to monitor effects at the individual cell level. Such approaches require development and maintenance of highly sophisticated equipment and analysis tools rendering strategies time consuming and expensive. Thus, alternative approaches to quantify GPCR-induced second messenger signals are still needed.

Here we focused on cAMP as a pivotal component of cellular signaling [37]. Typically, quantification of cAMP concentrations is performed using cytosolic extracts from cells or tissue. Such extracts can be applied, e.g., to high performance liquid chromatography (HPLC) and the cAMP concentration is calculated from a calibration curve obtained with standards of known concentration (e.g., [30]). However, mainly due to chromatography run-times this approach faces drawback especially when large sample numbers have to be handled. A reasonable alternative to HPLC separation is to apply either competitive binding assays to cAMP-binding proteins or cAMP-directed antibodies. Typically measurements are performed either with [^3^H]- or [^125^I]-cAMP as competitors [7, 9, 13]. Both approaches have been successfully and frequently applied in the last decades. The sensitivity of these assays allowed detection of fmol to μmol cAMP amounts. However, assay procedures typically require hours or overnight incubations and special equipment for storage, handling as well as discarding radioactive material and waste. It is noteworthy, that radiolabeled components meanwhile have been replaced by fluorescently labeled compounds (see, e.g., [11]), yet, experimental duration remained unaffected.

The discovery and development of optogenetic sensors and tools (reviewed in: [33]) finally paved the way to a new era for determining the dynamics of cellular signaling molecules. Currently, a large repertoire of sensors is available to monitor and examine fluctuations of second messengers and these sensors can be directed on demand to or into sub-cellular compartments [34–36]. Typically, sensors contain one to two fluorescent moieties that change their fluorescent properties upon interaction with a specific ligand. In combination with high resolution fluorescence microscopy, sensors are applicable for cell-based assays, provided that the genetic material coding for the sensor was successfully introduced into the cell or tissue [14].

To overcome limitations in applying optogenetic sensors for quantifying cAMP amounts in cell or tissue extracts, we optimized an expression and purification protocol for the cAMP sensor Epac1-camps. Affinity- followed by size-exclusion chromatography resulted in protein preparations of high yield and purity. We transferred purified protein to 96 MWPs resulting in an assay format applicable for high sample throughput. The sensor’s performance at different potassium concentrations and pH values was evaluated by measuring its fluorescence emission in a plate reader. Notably, measurements were conducted 20 to 30 min after adding the cyclic nucleotides representing a tremendous improvement in saving experimental time compared to approaches like HPLC or competitive binding assays. Neither salt concentrations up to 300 mM nor pH values ranging between 6.3 and 8.5 affected the sensor’s sensitivity to cAMP. Nevertheless, a prerequisite to generating data sets and comparing results from independent series of experiments is to keep assay conditions constant with respect to salt type and concentration, pH values, as well as solvents used for re-constitution of the sensor. Interestingly, we observed binding of Epac1-camps to cGMP, yet, with almost 10-fold lower affinity. Since cellular cGMP concentrations are much lower than cAMP concentrations under physiologic conditions, a cGMP “contamination” most likely does not compromise the assay. Using extracts from cell cultures in which cAMP production had been induced in a GPCR-dependent fashion showed that the assay was well suited to calculate concentration-response relationships and to determine the GPCR’s EC_50_. Furthermore, purified protein could be transferred into 96 MWPs, dried, and re-constituted without losing the sensors’ sensitivity even after several weeks of storage at 4 °C. Therefore, we expect that the rather simple expression and purification protocol for Epac1-camps-His_6_ protein in combination with the protein’s sensitivity and stability – even after storage in dried form – and versatile measuring format in 96 MWPs will support applications striving for robust, reliable, sensitive, and fast results in determining concentrations of cyclic nucleotides.

## Conclusions

Evaluation and quantification of intracellular signaling molecules, like cyclic nucleotides, is frequently used in bio-pharmaceutical tests studying, e.g., ligand-dependent GPCR activities. Here we examined and established an experimental setup that allows determination of cAMP in lysates obtained from cell-based assays or tissue samples using a bacterially overexpressed and purified FRET-based sensor protein, i.e. Epac1-camps. A two-step purification protocol by Ni-NTA followed by SEC resulted in milligram amounts of functionally active sensor protein. Assay conditions were evaluated with respect to different salt concentrations and pH values. Comparable and valid data can be obtained once the assay conditions are kept constant. With a detection threshold of ≅0.15 pmol, the assay was sensitive enough to determine cAMP amounts in lysates obtained from approximately 250.000 cells in which cAMP production was induced by GPCR activation. Notably, the protein could be desiccated and reconstituted in 96 MWPs without loss of functionality and sensitivity. In comparison to currently available cyclic nucleotide detection assays that require several hours of handling, stable fluorescence measurements were achieved within 30 min upon adding samples to Epac1-camps suggesting that the assay is suitable for routine and high throughput analyses.

## Methods

### Cloning an Epac1-camps construct for bacterial expression

For protein expression in *E.coli* cells the pcDNA3 plasmid containing the Epac1-camps encoding cDNA [27] was modified by PCR by adding a sequence coding for a hexa-histidine tag (His_6_-tag) at the C-terminus. The Epac1-camps construct was cut into EYFP- and ECFP-encoding fragments by *Hind*III/*Xba*I and *Xba*I/*Bam*HI, respectively, to prevent unspecific primer binding. Fragments were used as templates for individual PCR reactions to introduce a suitable restriction site to the 5′-end (*Nde*I) and a His_6_-tag followed by a stop codon and a *Bam*HI restriction site to the 3′-end. The following primers were used for amplification:

N-term (fwd) 5′-AAAACTCGAGCATATGGTGAGCAAGGGCGAGGAG,

N-term (rev) 5′-GCTCACTCTAGATTCCAGCCGCATGGTCTT,

C-term (fwd) 5′-GGAATCTAGAGTGAGCAAGGGCGAGGA, and C-term-His_6_ (rev)

5′-TTTTGGATCCCTAATGATGGTGATGGTGATGCTTGTACAGCTCGTCCATGCC.

For amplification the KOD Hot Start DNA Polymerase (Merck, Darmstadt, Germany) was used according to the supplier’s protocol. Template DNA was initially denatured for 5 min at 94 °C, followed by 20–30 repetitive cycles consisting of 1 min denaturation at 94 °C, and 1 min primer hybridization to the template. Elongation was performed at 72 °C for 45 s. Hybridization was performed at the melting temperature (T_m_) of the primer with the lower T_m_ for 1 min. PCR products were digested with restriction enzymes *Nde*I/*Xba*I for the EYFP- and *Xba*I/*Bam*HI for the ECFP-encoding fragment. After agarose gel purification, fragments were ligated into pET11a vector (Novagen, Merck Chemicals, Nottingham, UK) and sequenced.

### Expression of recombinant proteins in *E.coli*

Overexpression of Epac1-camps-His_6_ protein was performed in *E.coli* strains BL21(DE3)-pLysS (Merck) and BL21(DE3)-CodonPlus-RIL (Stratagene/Agilent, Santa Clara, CA, USA) that had been transformed with the recombinant plasmid (pET11a-Epac1-camps-His_6_). As these strains carry a chloramphenicol resistance, they were cultivated in antibiotic containing media. 500 ml LB media containing chloramphenicol (50 μg/ml) and ampicillin (100 μg/ml) were inoculated with an *E.coli* overnight culture and cultivated at 37 °C in a shaker (Unitron; Infors, Bottmingen, Switzerland) until an OD_600_ between 0.4 and 0.6 was reached. Protein expression was induced with 1 mM isopropyl-β-D-thiogalactopyranosid (IPTG). Induced bacterial cultures were grown either at 37 °C for 4 h, at 30 °C overnight, or at 20 °C overnight. Cells were harvested by centrifugation (Sorvall Evolution RC, SLA 3000 rotor, 5000 g, 15 min, 4 °C), snap frozen in liquid N_2_ and stored at − 80 °C.

### Ni-NTA purification of Epac1-camps-His_6_

All solutions were supplemented with cOmplete™ EDTA-free Protease Inhibitor Cocktail Tablets (Sigma-Aldrich/Merck, Darmstadt, Germany). The bacterial cell pellet was re-suspended in NPI-20 buffer (50 mM NaH_2_PO_4_, 300 mM NaCl, 20 mM imidazole, pH 8.0; 3 ml/g pelleted cells). Prior to an incubation step for 30 min on ice, lysozyme (1 mg/ml) and DNase I (≈ 0.5 mg; #A3778; Applichem, Darmstadt, Germany) were added. The suspension was sonicated 10 times for 15 s at 40% amplitude (Branson Sonifier, model W-450D, tapered microtip; Branson, Danbury, CT, USA) followed by centrifugation (Sigma 2 K15, 10,000 g, 45 min, 4 °C). The supernatant was used for purification of the Epac1-camps-His_6_ fusion protein via Protino® Ni-NTA Agarose (Macherey & Nagel, Düren, Germany). 1 ml of Ni-NTA agarose was transferred to a 50 ml reaction tube and equilibrated with 10 bed volumes of NPI-20 buffer. The matrix was pelleted by centrifugation (Sigma 3-16 K, 500 g, 5 min, 4 °C). The supernatant containing Epac1-camps-His_6_ was added to the matrix and agitated on a shaker for 30 min at 4 °C. The sample was centrifuged (Sigma 3-16 K, 500 g, 5 min, 4 °C). The supernatant was removed and the matrix was suspendend in 10 bed volumes NPI-30 buffer (50 mM NaH_2_PO_4_, 300 mM NaCl, 30 mM imidazole, pH 8.0). The matrix was collected by centrifugation (Sigma 3-16 K, 500 g, 5 min, 4 °C) and the washing step was repeated. The matrix was re-suspended in 5 ml NPI-30 buffer and transferred into a Poly-Prep® Chromatopraphy Column (Bio-Rad, Munic, Germany). Finally, the recombinant protein was eluted with 5 bed volumes NPI-150 buffer (50 mM NaH_2_PO_4_, 300 mM NaCl, 150 mM imidazole, pH 8.0). Aliquots of all purification steps were kept for further analysis.

### Size exclusion chromatography (SEC)

For further purification of the recombinant protein, size exclusion chromatography (SEC) was employed. A HiLoad 16/600 Superdex 200 column (GE Healthcare/Merck, Darmstadt, Germany) was used and operated with an ÄKTA chromatography system (GE Healthcare/Merck). Prior to sample application, the column was washed with two column volumes H_2_O and equilibrated with two column volumes SEC-buffer (150 mM NaCl, 50 mM Tris/HCl, pH 7.4). The Epac1-camps-His_6_ containing eluate obtained from the Ni-NTA matrix was concentrated to a volume of 1 ml with Amicon® Ultra Centrifugal Filters (Merck; cut-off 50,000 Da) and applied to the SEC column. Chromatography was performed with SEC-buffer at a flow rate of 1 ml/min and fractions of 1 ml were collected. Fractions containing Epac1-camps-His_6_ were pooled, concentrated as described above, snap frozen in liquid N_2_ and stored at − 80 °C until further use.

### In vitro characterization of Epac1-camps-His_6_

In order to examine the functionality of Epac1-camps-His_6_, purified protein was assessed in 96 multi-well plates (MWPs; Black Cliniplates, #9502867, Thermo Scientific, Dreieich, Germany) using a plate reader (Fluostar Omega, BMG Labtech, Ortenberg, Germany). Protein was diluted to a concentration of 0.7 μM in IS buffer (135 mM K-gluconate, 12 mM NaHCO_3_, 4 mM KCl, 0.8 mM MgCl_2_, 10 mM HEPES, pH 7.4) and 90 μl of the sample was added to each well of a 96 MWP. The basal fluorescence emission was determined upon excitation at 430 nm (10 flashes/well) and recording the emission at 475 nm (ECFP) and at 530 nm (EYFP) over 5 cycles. The time for measuring the whole MWP (i.e., one cycle) was 41 s. A concentration series of cAMP and cGMP was added resulting in final concentrations ranging from 10^− 9^ to 10^− 4^ M. After incubation for 30 min at room temperature, fluorescence emission was registered and the EYFP/ECFP ratio (R) was normalized to the ratio of the basal fluorescence prior to cAMP or cGMP addition (R_0_). R/R_0_ values were plotted against cyclic nucleotide concentrations. EC_50_ values were determined with a four-parameter nonlinear regression using GraphPad Prism v5.04 (GraphPad, San Diego, CA, USA). Four- to eight-fold determinations were performed for each ligand concentration and all measurements were repeated independently at least two to three times. Two preparations of independently expressed and purified Epac1-camps-His_6_ protein were used.

### Cell-based assays inducing cAMP production

We used a cell line previously established in our group that constitutively expresses an octopamine receptor from *Drosophila melanogaster* (DmOctβ1R [2];) to induce cAMP production upon receptor activation by octopamine application. Receptor-encoding cDNA was stably transfected into human embryonic kidney (HEK293; purchased from ECACC, no. 85120602) cells and receptor-expressing cells were selected in the presence of geneticin. Cells were grown in M10/G418 medium (M10/G418 = MEM + Glutamax™; 10% (v/v) fetal calf serum; 1% (v/v) antibiotics/antimycotics; 1% (v/v) non-essential amino acids; 800 μg/ml geneticin (all from Gibco/Thermo Fisher Scientific, Darmstadt, Germany)). Cells were propagated in 9 cm petri dishes at 37 °C, 5% CO_2_ and ~ 95% relative humidity. For incubation with different octopamine concentrations (10^− 9^ – 10^− 4^ M), approx. 250.000 cells per well of 24 MWPs were used. Incubations were performed at 37 °C for 30 min in the presence of the phosphodiesterase inhibitor isobutylmethylxanthine (IBMX; final concentration 100 μM) in PBS. Quadruplicate determinations were performed for each ligand concentration and the experiments were independently repeated three times. Reactions were stopped by aspirating the test solutions and adding 0.5 mL of ice-cold ethanol (100%). After 1 h at 4 °C, lysates was transferred to Eppendorf cups and lyophilized. Samples were reconstituted in double distilled H_2_O. To determine the amount of cAMP produced, Epac1-camps-His_6_ protein (0.7 μM) was transferred into the wells of a 96 MWP. Basal fluorescence emission (475 nm (ECFP) and 530 nm (EYFP)) was determined upon excitation at 430 nm over 5 cycles (s.a.) in a plate reader (Fluostar Omega). Reconstituted probes were added to the sensor and incubated for 30 min at room temperature. Then, the EYFP/ECFP emission ratio (R) for each well was measured and normalized to the emission ratio of the basal fluorescence (R_0_) in each well. A calibration was performed with known concentrations of cAMP. Values of cAMP for each well were calculated from the calibration curve. Data (mean values cAMP (±SD)) were analyzed and used to generate concentration-response curves for octopamine with PRISM 5.04 software.

## Data Availability

All data and materials will be made available by the authors.

## Abbreviations

cAMP: Cyclic adenosine 3′,5′-monophosphate
cGMP: Cyclic guanosine 3′,5′-monophosphate
ECFP: Enhanced cyan fluorescent protein
EYFP: Enhanced yellow fluorescent protein
FRET: Förster resonance energy transfer
G418: Gentamycin
GPCR: GTP-binding protein-coupled receptors
HEK293: Human embryonic kidney cell line
IBMX: Isobutylmethylxanthine
IPTG: Isopropyl-β-D-thiogalactopyranosid
IS buffer: Intracellular solution buffer
MWP: Multi-well plate
Ni-NTA: Nickel-nitrilotriacetic acid
PBS: Phosphate-buffered salt solution
SEC: Size-exclusion chromatography
TE: Tris-HCl / EDTA buffer
